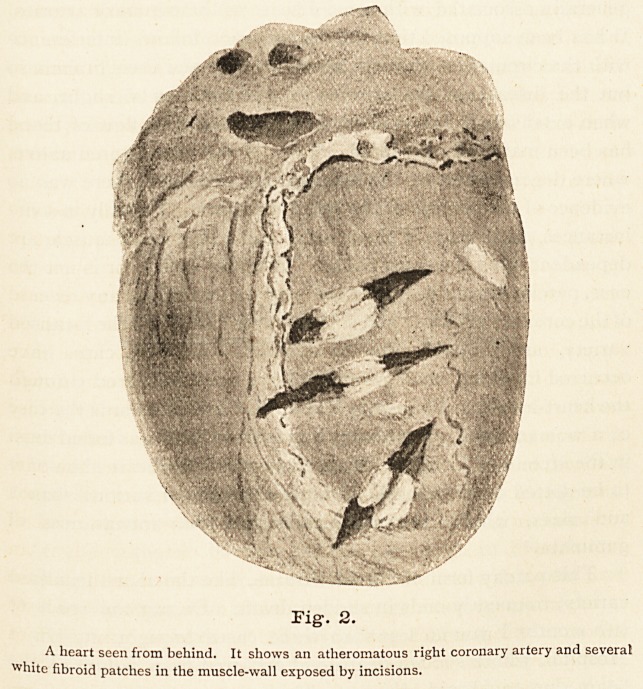# Two Cases of Fibroid Degeneration of the Myocardium

**Published:** 1895-12

**Authors:** Theodore Fisher

**Affiliations:** Pathologist to the Bristol Royal Infirmary


### TWO CASES OF FIBROID DEGENERATION OF THE MYOCARDIUM.

BY

THEODORE FISHER, M.D. Lond.,

*Pathologist to the Bristol Royal Infirmary.*

THE accompanying illustrations of examples of two cases of fibroid degeneration of the heart that have recently occurred in the *post-mortem* room of the Bristol Royal Infirmary may prove of some interest to those readers who have few opportunities of acquainting themselves with the appearances of morbid anatomy.

Fig. 1 is a sketch of a hypertrophied heart which shows considerable rounding of contour in the neighbourhood of the apex. The left ventricle is laid open by a longitudinal incision, and two apertures have been cut in its anterior wall. This incision and these apertures show how greatly the diameter of the wall of the ventricle differs at the apex and the base. At the base it is much thickened, but at the apex greatly thinned,

**Fig. 1.**

A heart seen from the left, showing a left ventricle of more or less globular shape, due mainly to fibroid atrophy and bulging of the wall in the neighbourhood of the apex.

the muscle substance having been replaced by fibrous tissue over an area about one inch and a half square. This is the main abnormal feature. Other less noteworthy details are visible in the mounted specimen, but are not seen in the sketch.

The two surfaces of the pericardium were universally adherent, but could be easily separated, except in the neighbourhood of the apex, where a small portion of the pericardium had to be left attached to the heart, and on opening the ventricle the greater part of the endocardium was seen to be of opaque milky-white appearance. All the valves were healthy and the coronary arteries were not diseased. The heart was removed from a woman aged 36, under the care of Dr. Shingleton Smith. There were indications of syphilis in the presence of scars of undoubtedly syphilitic nature on her forehead and in the neighbourhood of the knee-joints. There was also a small gumma in the left lobe of the liver, and scars of others were present as fissures in the right lobe. The case then appears to be fibroid degeneration of the myocardium following gummatous infiltration.

Fig. 2 illustrates the more common variety of fibroid degeneration. Three cuts on the posterior surface of the heart in the neighbourhood of the inter-ventricular septum open up white patches, which in the fresh state were glistening and tendinous in appearance. Two smaller white spots are seen on the surface of the heart marking the site of similar patches. The sketch endeavours to illustrate one other point. Running downwards over the ventricle a branch of the right coronary artery is represented, tortuous, thickened, and in places calcareous. The patches shown in the sketch were by no means the only fibroid spots present. There was a large one at the apex, others were in the septum, and the base of one of the musculi papillares was seriously affected. This specimen, which is an example of patchy fibroid degeneration associated with disease of the coronary arteries, was removed from a man, aged 56, who had been under the care of Dr. Prowse, and died of carcinoma of the stomach.

As indicated above, the first case is, with little doubt, an example of fibroid degeneration of the heart-wall following the presence of a gumma. In this case life had lasted sufficiently long for all definite traces of its gummatous origin to have disappeared; but death sometimes occurs while the local patch still presents the characters of a gumma, as in a case recorded by Dr. Newton Pitt, where a man, aged 28, died suddenly from rupture of the heart-wall at the diseased spot.[1] More commonly, however, life is prolonged until the destructive appearances of a gumma have disappeared and a fibroid area alone remains. The heart is of necessity weakened, not only generally but locally, by the fibroid area, which may yield before the strain of intra-ventricular blood-pressure, and give rise to an

Fig. 2.

A heart seen from behind. It shows an atheromatous right coronary artery and several white fibroid patches in the muscle-wall exposed by incisions.

aneurysm of the ventricular wall. Curiously enough, however, in spite of the destructive character of such a lesion the heart may give little indication that anything serious is wrong, until it suddenly fails, occasioning immediate death. This may occur

[1] *Tr. Path. Soc. Lond.*, 1891, xlii. 61.

whether a simple fibroid patch is present, or whether that patch has yielded so as to form an aneurysm. At a recent meeting of the Clinical Society, Sir Dyce Duckworth [1] recorded a case of sudden death of a man, aged 35, with aneurysm of the left ventricle. He mentions that the records of fourteen similar cases showed that sudden death occurred in eight instances.

The second case of which a sketch is given is an example of the patchy form of fibroid degeneration, which variety is generally associated with some disease of the coronary arteries. It has been supposed that the degeneration follows interference with the circulation through these arteries and their branches; but the disease of the arteries may sometimes be slight, and when extensive it is not always evident that the flow of blood has been materially hindered. Even in the case figured above, where degeneration of the arteries was extensive, there was no evidence of thrombosis of the small branches. Possibly in some instances the disease of the arteries and of the heart-muscle are dependent upon the same cause. Whether such is or is not the case, patchy fibroid degeneration may occur without any disease of the coronary arteries; and some of these cases, like the localised variety, are probably due to syphilis. At least, cases have occurred in which small gummata have been scattered through the heart-muscle. For example, Dr. W. Pasteur records the case of a woman, aged 30, probably a prostitute, who was found dead in the streets of London. Examination of the heart showed it to be dotted over with yellowish-grey patches of various shapes and sizes, which microscopically had the appearances of gummata.[2]

This patchy form of fibroid disease, like the more localised variety, frequently ends in sudden death. During the space of two months I saw no less than three cases brought into Guy's Hospital, where sudden death had occurred from failure of the heart due to fibroid disease. This experience, however, was exceptional, since only nine similar cases occurred during a period of ten years.

Some text-books speak of myocarditis following rheumatism

[1] *Brit. M. J.*, 1895, ii. 974.
[2] *Tr. Path. Soc. Lond.*, 1887, xxxviii. 103.

as a cause of fibroid disease of the heart. One can easily conceive this to be possible, since some increase of interstitial tissue in the myocardium may occur as a sequel of pericarditis the result of rheumatism. Possibly in rare instances rheumatism throws the force of its virulence wholly upon the myocardium, leading to the development of fibroid changes in the heart-wall, while the pericardium and valves remain healthy. One case I noticed on perusal of the Guy's Hospital *post-mortem* records, where death occurred in a boy, aged 16, from fibroid disease of the heart, that seemed to bear out such a view. Fibroid degeneration of the heart, however, rarely occurs at periods of life in which the heart is most susceptible to rheumatic poisons, and this cause of the disease cannot be a common one.

## Figures and Tables

**Fig. 1. f1:**
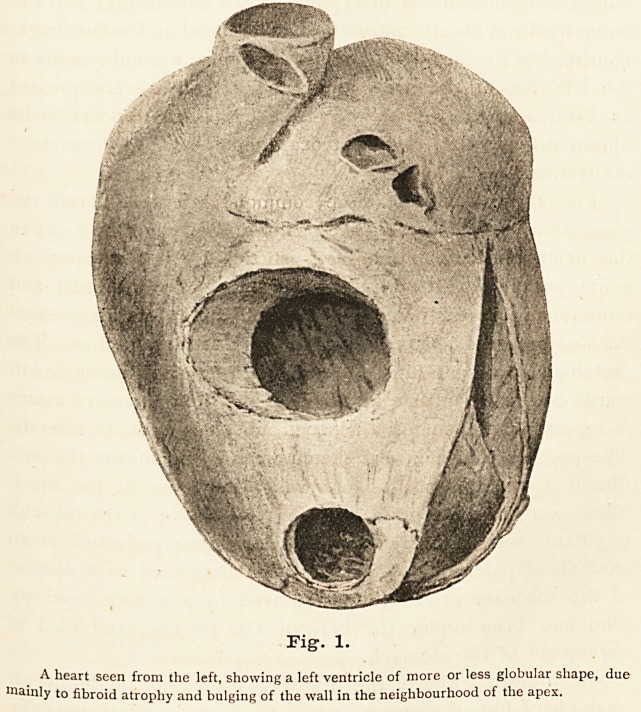


**Fig. 2. f2:**